# A laser‐Engraved Wearable Electrochemical Sensing Patch for Heat Stress Precise Individual Management of Horse

**DOI:** 10.1002/advs.202310069

**Published:** 2024-05-10

**Authors:** Yuxiang Pan, Xiaoyu Su, Ying Liu, Peidi Fan, Xunjia Li, Yibin Ying, Jianfeng Ping

**Affiliations:** ^1^ Laboratory of Agricultural Information Intelligent Sensing College of Biosystems Engineering and Food Science Zhejiang University Hangzhou 310058 P. R. China; ^2^ ZJU‐Hangzhou Global Scientific and Technological Innovation Center Zhejiang University Hangzhou 311215 P. R. China

**Keywords:** heat stress, laser engraved graphene, smart agriculture, sweat electrochemical analysis, wearable sensing patch

## Abstract

In point‐of‐care diagnostics, the continuous monitoring of sweat constituents provides a window into individual's physiological state. For species like horses, with abundant sweat glands, sweat composition can serve as an early health indicator. Considering the salience of such metrics in the domain of high‐value animal breeding, a sophisticated wearable sensor patch tailored is introduced for the dynamic assessment of equine sweat, offering insights into pH, potassium ion (K^+^), and temperature profiles during episodes of heat stress and under normal physiological conditions. The device integrates a laser‐engraved graphene (LEG) sensing electrode array, a non‐invasive iontophoretic module for stimulated sweat secretion, an adaptable signal processing unit, and an embedded wireless communication framework. Profiting from an admirable Truth Table capable of logical evaluation, the integrated system enabled the early and timely assessment for heat stress, with high accuracy, stability, and reproducibility. The sensor patch has been calibrated to align with the unique dermal and physiological contours of equine anatomy, thereby augmenting its applicability in practical settings. This real‐time analysis tool for equine perspiration stands to revolutionize personalized health management approaches for high‐value animals, marking a significant stride in the integration of smart technologies within the agricultural sector.

## Graphical abstract

An integrated wearable sensing patch for horse sweat analysis for heat stress is reported, including pH, K^+^, and temperature. The device integrates laser engraved graphene sensing electrode array, flexible signal processing module, and wireless communication circuit. The integrated system enables the early and timely assessment for heat stress, with high accuracy, stability, and reproducibility.

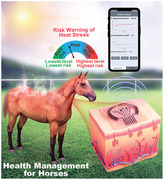

## 1 Introduction

Heat stress, a non‐specific physiological response, is frequently observed in animals.^[^ ^1^, ^2^, ^3^ ^]^ Normally, the animals present limited adaptability to environmental temperatures, rendering them vulnerable to heat stress when exposed to temperatures beyond their tolerance limits.^[^ ^4^, ^5^, ^6^ ^]^ Heat stress reveals adverse influence on the psychological and physiological of animals, and potentially endangering their health in husbandry breeding.^[^ ^7^, ^8^ ^]^ Especially, as a specific mammalian species, the horses are prone to heat stress due to their limited capacity for self‐temperature regulation, which can result in various physiological disruptions, including elevated body temperature, increased respiratory rate, dehydration, and impaired immune function.^[^ ^9^, ^10^, ^11^ ^]^ These effects contribute to a decline in equine exercise endurance, impaired performance, and an increased risk of heat‐related conditions.^[^ ^12^ ^]^ Up to now, a feasible and early management methods, focusing on precise individual management of heat stress, are still in significant demand.

A number of methods, such as physical environmental control and nutrient management, are conventionally adopted for heat stress management, while these methods may struggle to meet the growing requirements for precise management and ongoing monitoring.^[^ ^13^, ^14^ ^]^ Modern precision health management approaches for heat stress biomarkers, including conventional medical testing, typically involve invasive and time‐consuming procedures.^[^ ^15^ ^]^ Wearable electronics serve as non‐invasive devices that come into contact with the skin, enabling continuous and close monitoring of individual activity without causing any disruption or constraints to the organism's activities.^[^ ^16^, ^17^, ^18^, ^19^ ^]^ Among various kinds of analytes, the non‐invasive and accessible sweat contains numerous chemical components and indicators, including electrolyte, glucose, and lactate, which remarkably related to health and physiological status.^[^ ^20^, ^21^ ^]^ Hence, the utilization of sweat sensors for equine health management holds immense significance. Particularly, the level of potassium ion (K^+^) in sweat is regarded as a significant indicator of health, and the dehydration and muscle cramps can be appeared with low‐level K^+^.^[^ ^22^ ^]^ Another crucial parameter for sweat analysis is pH, which reflects the metabolism and homeostasis level of body.^[^ ^23^ ^]^ In addition, the metabolite levels in sweat are closely related to temperature fluctuation. Therefore, multiple analysis of sweat is meaningful. Synchronously, electrochemical method, characterized by high selectivity, rapid responses and affordable costs, is an ideal platform for real‐time analysis.^[^ ^24^, ^25^, ^26^ ^]^ Considering that, the synergy between electrochemical wearable sensors and sweat analysis, is hopefully providing a reasonable and sound answer for heat stress of horse.

Traditional silicon‐based wearable sensors face real‐analysis challenges, such as deployment and exhibit time delays, and requiring complex manufacturing processes and expensive microfabrication equipment. Fortunately, the unique advantages of flexible devices can efficiently alleviate these issues.^[^ ^27^, ^28^ ^]^ Crespo et al.^[^ ^29^, ^30^ ^]^ proposed novel wearable potentiometric ion sensors based on screen‐printed electrodes and successfully applied them to human sweat analysis, in which the flexible device presents excellent performance, affordable prices, and scalable manufacturing capabilities. Recently, a promising manufacturing technique, CO_2_ laser engraving, enables rapid pattern engraving and process optimization, has garnered significant interest.^[^ ^31^ ^]^ Laser‐engraved graphene (LEG) is typically obtained by CO_2_ laser engraving, revealing prominent advantages in scalability and customization.^[^ ^32^ ^]^ Flexible LEG‐based sensors are able to bring confident readout, benefitting from high conductivity and electromechanical stability, and reasonably earn more concerns in wearable biosensing field.^[^ ^33^, ^34^, ^35^, ^36^ ^]^ Particularly, LEG‐based wearable sweat sensors normally focus on electrolytes and metabolites monitored via ion selective sensors or enzyme electrodes, which can be diversely modified and realize simultaneous monitoring.^[^ ^37^, ^38^ ^]^

**Figure 1:**
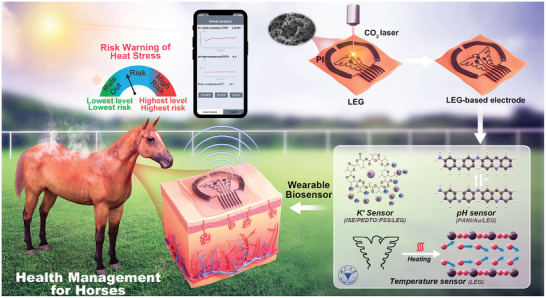
Schematic diagram of LEG‐based wearable sensor, in which the K^+^, pH, and temperature of the horse sweat for heat stress are well monitored by wireless devices.

Herein, a LEG‐based continuous sweat analysis patch, with attractive feature of multiple real‐time and wireless analysis, is introduced for pH, K^+^ and temperature non‐invasive monitoring in horse sweat (**Figure** **1**). The LEG‐based sensor is large batch manufactured on a flexible polyimide (PI) film, similar to flexible printed circuit, featuring a low‐cost pattern. Synchronously, LEG‐based device is regarded as an ideal platform for iontophoretic sweat induction, flexible signal processing module and wireless communication circuit. Profiting from ion‐selective electrodes (ISE) and pH‐sensitive materials, the sensitive and specific monitoring are well realized, and the electrochemical signal is further amplified. Modifications, mechanisms and advances of wearable patch is subsequently validated with morphological and electrochemical methods. Finally, with a Truth Table capable of logical evaluation, the proposed method was confirmed in real‐time sweat analysis for heat stress management of horses.

## 2 Results and Discussion

### 2.1 Wearable Sensing Patch Design and Fabrication

**Figure 2:**
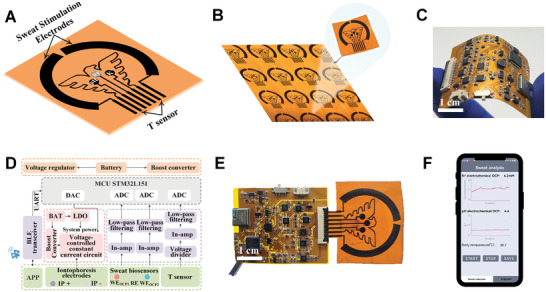
A) Function description of LEG‐based electrode. B) Large‐scale production of LEG sensor arrays and disposable single LEG‐based array. C) Flexible design of FPCB. D) Diagram of signal transduction, processing, and wireless transmission from the sensor to the user interface. E) Flexible wearable LEG‐based sensing patch can be simply attached to a designed FPCB. F) Wearable patch wirelessly connects to a custom‐developed mobile APP.

The flexible wearable sensor patch was orderly comprised of a PI substrate layer and a LEG‐based electrode, and they could be well manufactured and modified. During sweat analysis, the patch primarily consisted of four components: two ionophoretic electrodes, a reference electrode, a pH electrode, a K^+^ working electrode, and a temperature sensor (**Figure** **2**A). All functional modules of the flexible electrode were manufactured using LEG technology (Figure 2B), which offered a substantial surface area and low sheet resistance of 150 Ω sq^−1^. The level of sweat evaporation and diffusion in areas was well slowed down by the covered patch, ensuring continuous contact between the electrode and sweat. The prepared wearable sweat sensing patch exhibited the advantages of lightweight and compact. Particularly, the purchase of multiplex sensor fabrication was well controlled at dollar level. For chemical sensing, each module was individually controlled by a pre‐programmed program. The potential response was amplified by a potential in‐amplifier and converted to voltage, which was read by an analog‐to‐digital converter (ADC). The sweat sensing circuit was comprised of a programmable current source for the delivery of iontophoretic current and a protective circuit, which established an upper limit for the iontophoretic current as a safety mechanism (Figure 2C and D). A wearable sensing patch could expediently affix to a reusable flexible printed circuit board (FPCB) for electrochemical data acquisition, wireless communication, and programmable voltage modulation (Figure 2E). In particular, the LEG‐based wearable patch and FPCB exhibited appropriate dimensions (Figures S1 and S2, Supporting Information). Real‐time signal processing and wireless communication with the user interface were performed on‐site through Bluetooth, enabling users to monitor the health status of animals with smartphones (Figure 2F).

### 2.2 Characterizations of Wearable LEG‐Based Sensing Patch

**Figure 3:**
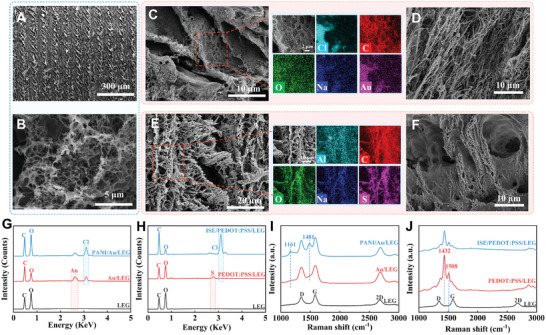
SEM images of A) bare LEG and B) enlarged structure. C) SEM and elemental mapping images of Au/LEG. D) SEM image of PANI/Au/LEG. E) SEM and elemental mapping images of PEDOT:PSS/LEG. F) SEM image of ISE/PEDOT:PSS/LEG. G) EDS spectra of bare LEG, Au/LEG, and PANI/Au/LEG. H) EDS spectra of bare LEG, PEDOT:PSS/LEG, and ISE/PEDOT:PSS/LEG. I) Raman spectra of bare LEG, Au/LEG, and PANI/Au/LEG. J) Raman spectra of bare LEG, PEDOT:PSS/LEG, and ISE/PEDOT:PSS/LEG.

Morphologic characteristics of LEG, with different modification, were observed with scanning electron microscope (SEM). Distinct and regular scuffing of LEG surface was clearly identified in **Figure** **3**A. Using a zoom with higher magnification, the LEG presented representative 3D graphene framework with a multilayered porous structure (Figure 3B). Normally, the focused laser beam induced localized high temperatures on the PI film surface, which contribute to carbonization and graphitization on the PI substrate. The formation of the porous structure could be attributed to the combustion of certain carbon content during the graphitization process.^[^ ^39^ ^]^ With HAuCl_4_ electrodeposition, the Au nanoparticles were equably plated on the LEG (Figure 3C). In mapping elemental exploration, the Cl, C, O, Na, and Au were explicitly observed, indicating successful preparation of Au/LEG. After polyaniline (PANI) modification, the PANI chains exhibited a particular 3D network structure and consistent distributed on the Au/LEG, and thus well formed PANI/Au/LEG (Figure 3D). In particular, 3D PANI is constructed from numerous interconnected branched nanofibers of PANI, featuring an abundance of voids distributed throughout its structure. These interconnected branched nanofibers provide continuous conductive pathways, and the large pores between the solid networks form smooth channels for the transport of hydrogen ions. Compared with other granular or blocky materials, the 3D structure could efficiently facilitate charge transfer. Additionally, elemental mapping of PANI/Au/LEG were shown in (Figure S3, Supporting Information). During ISE fabrication, poly(3,4‐ethylenedioxythiophene):poly(styrene sulfonate) (PEDOT:PSS) was employed as a stable conductive substance for potentiometric ion sensors and was applied in LEG modification. As depicted in Figure 3E, PEDOT:PSS formed a thin conductive layer on the LEG surface. The electro polymerization of PEDOT:PSS was further investigated through the mapping analysis, which revealed the uniform dispersion of Al, C, O, Na, and S. Subsequently, the modification of ISE leaded to a K^+^ selective membrane coverage on entire working electrode surface, where a certain thickness was possessed (Figure 3F).

Energy dispersive spectrum (EDS) spectra were applied to suggest the elemental variation of LEG, Au/LEG, PANI/Au/LEG, PEDOT:PSS/LEG, and ISE/PEDOT:PSS/LEG. For pH electrode fabrication, the bare LEG exhibited characteristic C and O element peaks. With the electrochemical deposition of HAuCl_4_·_3_H_2_O on LEG, a distinct Au peak was observed on the Au/LEG electrode. Following the polymerization of PANI, an obvious increase in Cl was observed, indicating the successful formation of the PANI/Au/LEG electrode (Figure 3G). For the detection of K^+^, the LEG electrode was modified sequentially with PEDOT:PSS and ISE, and the EDS results showed the presence of S and Cl elements on the modified electrode surface. As the amount of PEDOT:PSS and ISE modification increased, the content of C and O elements on the electrode surface also showed reasonable changes (Figure 3H). Moreover, the preparation of reference electrode was also investigated with element mapping and EDS. As illustrated in (Figures S4 and S5, Supporting Information), Ag and Cl elements were respectively observed on the Ag/LEG and polyvinyl butyral (PVB)/Ag/LEG, confirming the successful fabrication of reference electrode.

Structural characteristics of LEG‐based electrode was explored with Raman spectra. Bare LEG displayed three distinct bands, including D‐band (1344 cm^−1^), G‐band (1575 cm^−1^), and 2D‐band (2670 cm^−1^), which was respectively represented the structural defects created by sp3 hybridized carbons, E2g phonon vibration of graphitic carbon, and the resumption of sp2 hybridized carbons. Normally, the values of the I_2D_/I_G_ ratio indicated the degree of graphitization‐photoreduction and the transformation into a graphene‐like structure, while the I_D_/I_G_ represented the photoinduced defect density.^[^ ^40^ ^]^ With Au modification, the I_2D_/I_G_ and I_D_/I_G_ were regularly increased. A reasonable explanation is that the Au nanoparticles hindered the accumulation of graphene sheets, and a large number of defects appeared (Figure 3I). After PANI overlaying, two new Raman peaks (1161 and 1481 cm^−1^) was distinctly surveyed, indicating that the PANI/Au/LEG was well formed. During the fabrication of the K^+^ sensing electrode, two characteristic peaks appeared between the G and D peaks at 1432 and 1508 cm^−1^ after PEDOT:PSS electrodeposition, which attributed to the symmetric and asymmetric stretching vibrations of C_α_═Cβ in PEDOT:PSS.^[^ ^41^ ^]^ Meanwhile, the 2D peak of LEG was reasonably covered due to the π–π interaction between LEG and PEDOT:PSS.^[^ ^42^ ^]^ As expected, with ISE addition, the characteristic peaks were regularly restrained (Figure 3J). Additionally, the manufacture of reference electrode was also explored with Raman spectroscopy. With Ag electrodeposition, the value of I_D_/I_G_ was obviously changed (Figure S6, Supporting Information). In particular, the pure PI substrate hardly showed any Raman spectral peaks (Figure S7, Supporting Information). Finally, the crystal structures of bare LEG and Ag/LEG were investigated using an X‐ray diffractometer (XRD) (Figure S8, Supporting Information). Bare LEG presented characteristic diffraction peaks at 26.1° and 45.4°, which were assigned to crystal faces of (002) and (100). For Ag/LEG, the representative crystal faces appeared at (111), (200), (220), and (311). Above results conformably revealed the successful preparation of PANI/Au/LEG, ISE/PEDOT:PSS/LEG, and reference electrode.

### 2.3 Electrochemical Performance Evaluation of the Wearable Sensing Patch

**Figure 4:**
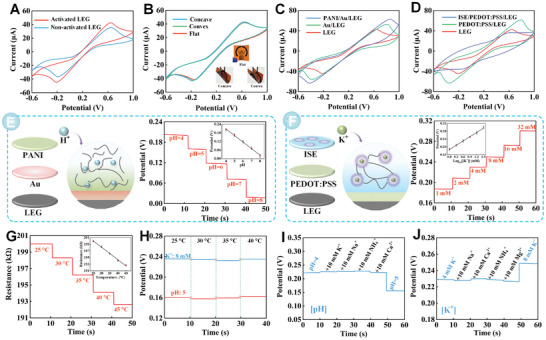
A) CVs of activated and non‐activated bare LEG. B) CVs comparison of different bending stress modes, including flat, convex, and concave, respectively. C) CVs curves for stepwise modifications of bare LEG, Au/LEG, and PANI/Au/LEG. D) CVs curves for stepwise modifications of bare LEG, PEDOT:PSS/LEG, and ISE/PEDOT:PSS/LEG. All CVs curves are measured in 5 mM K_4_[Fe(CN)_6_] and 0.1 M KCl, and the commercial Pt electrode and Ag/AgCl electrode are respectively as counter and reference electrode. OCP responses of LEG‐based sensors for E) pH, F) K^+^, and G) temperature determination. Corresponding calibration plots of the sensors are presented in (E) to (G). H) OCP responses of 8 mM K^+^ and pH = 5 in different temperature. Selectivity of the LEG‐based I) pH and J) K^+^ sensors.

For wearable sweat sensing patch, the analysis performances were evaluated with different electrochemical process. Typically, it is crucial to carry out electrochemical cleaning process before experimentation. Consequently, cyclic voltammetry (CV) was performed on the bare LEG for 20 cycles in 0.5 M HCl, where the potential window and scan rate were set as −0.1–0.9 V and 0.1 V s^−1^, respectively. As depicted in **Figure** **4**A, the CV peak currents significantly increased after HCl cleaning, indicating enhanced electrode exposure of active sites, which was conducive to electron transfer. In addition, a rising tendency in bare LEG redox signals were obviously surveyed with gradual scan rates as 0.01–0.1 V s^−1^, well confirming linear relationship between square root of the scan rates and redox peak (Figure S9, Supporting Information). It revealed that good diffusion‐controlled process was appeared on bare LEG. As a wearable flexible patch, the flexibility of sensors is of paramount importance in practical applications. Therefore, the impact of bending stress under flat, convex, and concave conditions of the sensor was assessed with CVs in a 5 mM [Fe(CN)_6_]^4^ ^−^ solution containing 0.1 M KCl. As shown in Figure 4B, the CV peaks showed a similar variation tendency under different bending stresses, indicating that the prepared flexible sensor presented excellent mechanical stability. Subsequently, the modification processes of bare LEG, Au/LEG, PANI/Au/LEG, PEDOT:PSS/LEG, and ISE/PEDOT:PSS/LEG were compared with CVs. For bare LEG, symmetrical shapes and invertible redox peaks were well examined. Profiting from the abundant surface area of AuNPs, the efficiency of electrochemical reactions can be reasonably raised, and they can also provide more active sites and increase the electron transport rate.^[^ ^43^ ^]^ Therefore, with AuNPs electrodeposition, the peak currents were signally promoted, and a solid conductive layer was formed on the LEG‐based electrode surface. Subsequently, a continuing upward tendency was observed in PANI/Au/LEG (Figure 4C). A reasonable explanation is that the three‐dimensional PANI presented abundant porous structure, which is conducive to the increment of the electroactive area for H^+^, and thus lead to the acceleration of the electron ship speed.^[^ ^44^ ^]^ Therefore, a sensitive pH sensor was well manufactured. For ISE sensor, an incremental tendency was observed with PEDOT:PSS modified, which attribute to large surface area and excellent conductivity. Oppositely, redox peaks showed proper decrement after dropping of ISE, since the covered membrane blocked the electrons transmission (Figure 4D). Moreover, the performance of the reference electrode was compared by performed open‐circuit potential (OCP). As shown in (Figure S10A, Supporting Information), the Ag/AgCl modified LEG showed a smaller potential difference than the bare LEG for commercial reference, indicating that the modified Ag/AgCl was feasible. Furthermore, the stability of the two reference electrodes, including LEG‐Ag/AgCl and LEG‐Ag/AgCl‐PVB, were compared at different ion levels. The results showed that the LEG‐Ag/AgCl‐PVB presented outstanding ion stability (Figure S10B, Supporting Information).

The performance of sweat sensor was separately explored with different measuring level, including pH, K^+^, and temperature. As seen in Figure 4E, the OCP variation of pH sensors with physiologically relevant level of 4–8. With the increase of pH, the OCP potential showed a downward trend and presented a good linear relationship, with a sensitivity of 63.8 mV dec^−1^. Profiting from the ordered three‐dimensional chain structure within the PANI film, the efficient transport of H^+^ was facilitated. Similarly, the continuous OCP monitoring of K^+^ was based on a potentiometric ISE, where the binding between K^+^ and ionophore results in a potential variation. With a sensitivity of 60.3 mV dec^−1^, a good linear equation was well established between K^+^ levels and potential response (Figure 4F). Commonly, Ag/AgCl electrode was employed as reference electrodes for ISE. Particularly, with diverse ion strengths, the reference electrodes coated with PVB well maintained reference potential in sweat samples, and thus declining interactions between ion‐selective sensors.^[^ ^45^ ^]^ Normally, LEG exhibited unique performance in the fabrication of resistive physical sensors. With the ascent of temperature, the electron‐phonon scattering and thermal velocity of electrons in the LEG sandwiched layers was increased, and thus the conductivity was promoted (Figure 4G).^[^ ^46^ ^]^ Furthermore, the influence of temperature on sweat sensors was also evaluated. As depicted in Figure 4H, the maximum potential variation of 8 mM K^+^ and pH = 5 was calculated as 2.8% and 2.5%, indicating the unobvious potential variation with increasing temperature (25–40 °C). Thus, the impact of temperature on the sensor could be negligible. Reproducibility exploration of pH and K^+^ sensors were described in (Figure S11, Supporting Information). Five sweat sensors manufactured from different batches exhibited similar potential responses, and the fluctuation of pH and K^+^ sensors were well controlled in 6.3% and 4.2%, indicating acceptable reproducibility. A number of interfering ions, such as Na^+^, NH^+^, Ca^2+^, and Mg^2+^, might employ potential influence on analyses. Benefiting from the selective recognition elements, the response of the pH and K^+^ sensors did not exhibit significant changes upon the addition of interfering ions, indicating the proposed sensor exhibited excellent anti‐interference capability (Figure 4I,J). Moreover, the potential variations in the pH and K^+^ sensors before and after bending were investigated (Figure S12, Supporting Information). After 400 bending cycles, the sensor remained stable, indicating the sensor presented satisfactory durability. Primary features of this work, as well as related valuable studies from other scholars, were summarized in Table S1 (Supporting Information).

### 2.4 Heat Stress Evaluating of Horse Using Wearable Sensing Patch

**Figure 5:**
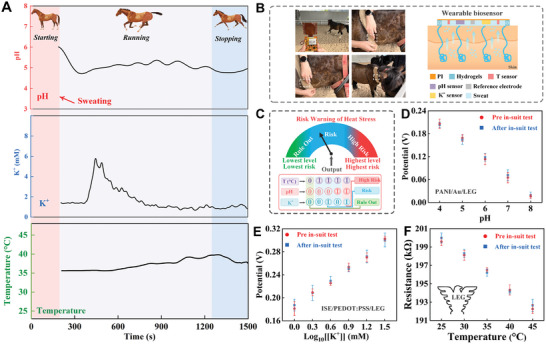
A) Real‐time monitoring of pH, K^+^ and temperature levels with various running states using wearable sensing patch. B) The diagram of wearable module on the surface of horse skin. C) The Truth Table to identify different risks of heat stress. Response of D) pH, E) K^+^, and F) temperature sensors in the wearable sensing patch before and after testing.

The designed wearable patch is intended as a wireless real‐time monitoring platform for the horse health management. Real‐time sensed signals were recorded by the FPCB, and further amplified and converted into digital signals. Processed data was wirelessly transmitted via a Bluetooth module to a mobile application (Video S1, Supporting Information). As depicted in **Figure** **5**A, the pH, K^+^ and temperature levels from horses sweat in running states were recorded, with the wearable patch securely fixed on the horse with elastic bandage. Particularly, the wearable module was applied to the surface of horse skin as shown in Figure 5B. Excellent compatibility between the sensors and equine skin facilitated stable and reliable real‐time monitoring during body tests. During the initial phase of running, the sensors did not operate steadily due to insufficient sweat to establish the dual‐electrode configuration of the electrochemical cell. Once an adequate level of sweat was achieved, the pH and K^+^ levels in the sweat was well stabilized. Throughout continuous running, the pH continually decreased and reached stability. A reasonable explanation is that the skeletal muscle cells in the body fail to acquire sufficient oxygen during intense exercise, and thus leading to substantial lactate production.^[^ ^47^ ^]^ For the K^+^ levels, the concentration initially increased and then decreased, which maintaining within normal physiological levels. Moreover, the temperature levels presented upward tendency with exercise. Above results indicated the nutritional supplementation was needed if the ion levels fall below a certain threshold during prolonged health monitoring.

The utilization of a multi‐index approach for assessing heat stress in horses has been demonstrated to produce a synergistic effect, thereby enhancing the precision of management strategies. Normally, the Truth Table represents a logical operation of an output signal, which is then combined to produce an unsigned binary counting method (“0” or “1”), and expresses in tabular format. When all output parameters are logical “1”, the output value is logical “1”, and the event is judged to be true, and thus the Truth Tables are well suited for multi‐parameter evaluation.^[^ ^48^ ^]^ Extensively, heat stress effect in animals is directly related to temperature, and when the temperature exceeds 40.5 °C, there is a tendency to occur heat stress.^[^ ^49^ ^]^ For K^+^ and pH in sweat, the normal range was remained at 1–24 mM and 4∼8, respectively.^[^ ^50^, ^51^ ^]^ The unbalance of electrolyte levels in sweat can lead to alteration in physiological behavior, which can impact health of horse. Considering the high concentration of electrolytes in horse sweat, the threshold values for the key indicator were established as 40.5 °C (body temperature), 4 (pH), and 3 mM (K^+^). Therefore, a Truth Table capable of logical evaluation was skillfully designed to identify different risks of heat stress (Figure 5C). Profiting from the advantages of multiple analysis of the wearable patch, the modification strategy could accurately predict the state of animal heat stress. The obtained analyte levels could be succinctly led to the Truth Table, with logical judgements of “0” and “1”. According to cut‐off levels of temperature (>40.5 °C), pH (<4) and K^+^ (<3 mM) levels, the state was interestingly categorized. The logical results were set as: “0/0/0” presented rule out heat stress; “1/0/0”, “1/0/1”, and “1/1/0” as heat stress risk; “1/1/1” as high risk of heat stress. The simple risk assessment of heat stress based on wireless wearable module can provide timely monitoring and treatment for animal, and further for early screening of health failure and prognosis assessment. Moreover, with iontophoretic electrodes, automatic sweat induction and monitoring could be realized during rest periods, which allowed for early management of horse health (Figure S13, Supporting Information). Furthermore, we examined the linear response before and after animal body testing. There were no significant fluctuations of pH (Figure 5D), K^+^ (Figure 5E), and temperature (Figure 5F), indicating reliable data for in‐body analysis. To verify the accuracy of the sensor, real sweat samples were collected and analyzed by this method and the standard method, and the results presented satisfactory consistency (Table S2, Supporting Information).

## 3 Conclusion

In summary, we develop a wireless wearable bioelectronic system, for multiplexed sweat monitoring on horse. The laser‐engraved multimodal sensors efficiently enable sweat inducing, sensitive sensing and multi‐channel vital signs sensing. A notable aspect is the integration of LEG‐based sensor arrays into wireless wearable technology, facilitating field signal processing, calibration, and wireless communication. During heat stress precise individual management of horse, the pH, K^+^ and temperature in sweat is precisely monitored with proposed wearable sensing patch. Reasonably, the integrated LEG‐based sensor arrays present desirable accuracy, stability and reproducibility. Furthermore, considering the matching degree between sweat‐sensing system and animal skin still depend on human interventions, subsequent programs, including the implementation of a smart micro‐fluidic system, are being considered to overcome such limitations. The recommended platform is considered a promising tool and provide a non‐invasive, continuous means, and guide personalized fitness management for horse. Given this, the proposed sensor reveals great potential in personalized health management for high‐value animals at smart agriculture.

## 4 Experimental Section

### Reagents

Reagents information was given in Section S1 (Supporting Information). Particularly, abbreviations of chemicals were described in this section.

### Preparation of LEG‐based Sensors

For LEG fabrication, the PI film was putted in a laser cutter with 50‐W CO_2_. Briefly, with the CO_2_ laser cutter was engraved to a PI film, the absorbed laser energy was availably converted into local heat, resulting in local high temperatures (>2500 °C). Particularly, the chemical bonds in PI network was reasonably broken and the carbon atoms was undergone a thermal recombination, and thus a sheet graphene structure was interestingly generated. Under optimal conditions, the power, speed, and points per inch of laser cutter were set as 8%, 14%, and 750, respectively. As control, a designed LEG sensor substrate was well prepared. In reference electrode preparation, the Ag was firstly modified on a LEG substrate by multi‐current electrodeposition in a plating solution containing 0.25 M AgNO_3_, 0.75 M Na₂S₂O₃, and 0.5 M NaHSO_3_. The electrodeposition procedure was respectively selected as −0.01 mA for 150 s, −0.02 mA for 50 s, −0.05 mA for 50 s, −0.08 mA for 50 s, and −0.1 mA. For PVB/Ag/AgCl/LEG electrode fabrication, the prepared 0.1 M FeCl_3_ was further dropped on the Ag surface for 30 s. After that, the 79.1 mg PVB and 50 mg NaCl was dissolving in 1 mL methanol under intense stirring conditions, and the 6 µL prepared reference cocktail was dropped on the Ag/AgCl/LEG electrode and dried overnight. Particularly, the shape design of the temperature sensor was inspired by the school emblem of Zhejiang University.

### Modification of Sweat Induce Working Electrodes

The sweat induce electrode was fabricated according to iontophoresis method. For anode gel manufacture, agarose (3%, w/w) was added into de‐ionized water and heated to 250 °C under constant stirring. After the mixture was completely dissolved, the solution was cooled to 165 °C, and then the 1% carbachol was add into above compound. After that, the cooled hybrid was slowly flowed into pre‐made cylindrical until fully solidified at 4 °C. Comparing with anode gel, the cathode gel was similarly prepared, while the carbachol was instead by NaCl (1%, w/w).

### Modification of pH Working Electrode

The pH sensor was prepared by electrodepositing a sensitive Au and PANI film on bare LEG electrode. Briefly, the LEG electrode was electrochemically cleaned in 0.5 M HCl by CV, where the scan cycle, potential window, and scan rate were separately set as 20, −0.1–0.9 V, and 0.1 V s^−1^. After that, −0.8 V potential was performed on cleaned LEG electrode for 60 s in 2.5 M NH_4_Cl solution containing 10 mM HAuCl_4_, forming Au/LEG. Subsequently, the prepared Au/LEG was immersed in 0.1 M aniline and 1 M HCl solution by CVs (−0.2–1.0 V) in 12 cycles, with an external Ag/AgCl reference and counter electrodes. In this case, the PANI film was equably modified, and well forming PANI/Au/LEG electrode for pH sensing.

### Modification of K^+^ Working Electrode

For K^+^ working electrodes preparation, the PEDOT:PSS) was firstly selected as ion‐electron transducer to diminish the potential drift of the ISE.^[^ ^52^ ^]^ In a solution containing 0.01 m EDOT and 0.1 m NaPSS, a constant current of 14 µA was applied on bare LEG surface for 10 minutes with an external Ag/AgCl reference and counter electrode, and the PEDOT:PSS was efficiently electrochemical deposited. Then, the K^+^‐selective membrane cocktail was prepared through mixed the composed of valinomycin (2%, w/w), DOS (64.7%, w/w), PVC (32.7%, w/w), and NaTPB (0.5%, w/w). After that, 100 mg membrane cocktail mixture was dissolved in 350 µL cyclohexanone. Finally, 6 µL K^+^ ion‐selective solutions were dropped on PEDOT:PSS/LEG, and the K^+^ detection electrode was well formed.

### Design of Hardware Circuit Software

The FPCB module comprised a necessary integrated circuit chips and a peripheral electronic component. Ion electrophoresis, signal processing, control, and wireless transmission circuits could be well realized in a programmable system. Entire system was built based on STM32L151 microcontroller, and equipped with analog circuits for sensor data acquisition and ion electrophoresis current control. When the microcontroller received user commands from the Bluetooth module via the Universal Asynchronous Receiver/Transmitter communication, it initiated sweat sensing and ion electrophoresis programs. The microcontroller controls the digital‐to‐analog converter (DAC) to set the control voltage for the current source via a serial peripheral interface. To optimize the design, a 100 µA current (≈2.6 µA mm^−2^) was adopted for surface ion electrophores on flexible patches. The sweat sensing circuit was OCP measurements and temperature sensing. The potentiostat circuit was comprised of control amplifiers and transimpedance amplifiers. A series voltage reference and a DAC were used to generate dynamic potential biases on the reference and working electrodes. Two instrumentation amplifiers (INA333, Texas Instruments) were employed for potential measurements, and a voltage divider was utilized for resistive temperature sensors. All analog voltage signals were acquired by the built‐in ADC channels in microcontroller, and transmitted via Bluetooth to the user device. The Bluetooth transceiver was connected to the microcontroller, enabling the system to communicate with a mobile phone. Through a mobile phone APP, users could well command different levels of ion electrophoresis current output and real‐time sensor data transmission.

### Horse Sweat Analysis

The wearable sweat sensor was attached on healthy horse (provided by Ai Chuancheng Equestrian Association, Hangzhou, China), and ran outdoors until the sweat was generated. After in situ determination, sweat samples were collected in centrifuge tube, and preparation for inductively coupled plasma mass spectrometry (ICP‐MS) and pH meter analysis. Sweat samples were collected in strict accordance with the protocol approved by the Experimental Animal Welfare Ethics Review Committee and not cause invasive damage to the horse.

### Apparatus for Characterizations

SEM (JSM‐7800 M, JEOL, Japan) was adopt to observed the surface morphology of modified LEG electrode. Universal laser system (PLS6MW, Zhongtian Juhe, China) was performed to form LEG substrate. Raman spectrum (LABRAM HR Evolution, HORIBA Jobin Yvon, France) was adopt to recorded the structural characteristics of LEG with a 532 nm laser. Crystal phases were explored by XRD (D8 ADVANCE, Bruker, Germany). Elemental compositions were analyzed with EDS (Octane SDD, AMETEK, USA). ICP‐MS (iCAP6300, Thermo Fisher, America) was adopted to verify the certified reading. Parameter analyzer (4200A‐SCS, Keithley, China) was used to recorded temperature response. Electrochemical analyzer (CHI‐660D, CH Instruments, China) and wireless multichannel electrochemical analyzer (WMEM‐8200A, ZHIXIN Tech, China) were utilized in electrochemical experiments. In electrochemical analysis, the LEG‐based electrodes as the working electrode, a platinum wire as the counter electrode, and a commercial Ag/AgCl electrode as the reference electrode. CV and OCP procedures were applied to explore stepwise modification and electrode stability of LEG sensors. The quantitative determinations of pH and K^+^ were obtain by OCP, where the time interval was set as 10 s.

## Conflict of Interest

The authors declare no conflict of interest.

## Author Contributions

Y.P and X.S contributed equally to this work. Conceptualization, methodology, writing‐original draft, and visualization were performed by Y.P and X.S. Formal analysis was performed by Y.L.. Data curation was performed by P.F. Data curation and investigation were performed by X.L. Writing‐review, project administration, and funding acquisition were performed by Y.Y. and J.P..

## Supporting information

Supporting Information

Supplementary Video S1

## Data Availability

The data that support the findings of this study are available from the corresponding author upon reasonable request.

## References

[1] G. N. Doering , I. Scharf , H. V. Moeller , J. N. Pruitt , Nat. Ecol. Evol. 2018, 2, 1298. 29942021 10.1038/s41559-018-0592-5

[2] P. A. Gonzalez‐Rivas , S. S. Chauhan , M. Ha , N. Fegan , F. R. Dunshea , R. D. Warner , Meat Sci. 2020, 162, 108025. 31841730 10.1016/j.meatsci.2019.108025

[3] Z. Guo , L. Lv , D. Liu , B. Fu , Trop. Anim. Health Pro. 2018, 50, 1203. 10.1007/s11250-018-1633-4 29948773

[4] I. B. Slimen , T. Najar , A. Ghram , M. Abdrrabba , J. Anim. Physiol. Anim. Nutr. 2016, 100, 401. 10.1111/jpn.12379 26250521

[5] S. Wasti , N. Sah , B. Mishra , Animals. 2020, 10, 1266. 32722335 10.3390/ani10081266 PMC7460371

[6] F. Xu , R. Li , E. D. von Gromoff , F. Drepper , B. Knapp , B. Warscheid , R. Baumeister , W. Qi , Nat. Commun. 2023, 14, 4176. 37443152 10.1038/s41467-023-39882-8 PMC10345090

[7] S. Gupta , A. Sharma , A. Joy , F. R. Dunshea , S. S. Chauhan , Animals. 2023, 13, 107. 10.3390/ani13010107 PMC9817836 36611716

[8] A. Nawab , F. Ibtisham , G. H. Li , B. Kieser , J. Wu , W. C. Liu , Y. Zhao , Y. Nawab , K. Q. Li , M. Xiao , L. L. An , J. Therm. Biol. 2018, 78, 131. 30509629 10.1016/j.jtherbio.2018.08.010

[9] R. J. Geor , L. J. McCutcheon , G. L. Ecker , M. I. Lindinger , J. Appl. Physiol. 2000, 89, 2283. 11090580 10.1152/jappl.2000.89.6.2283

[10] H. Kang , R. R. Zsoldos , A. Sole‐Guitart , E. Narayan , A. J. Cawdell‐Smith , J. B. Gaughan , Int. J. Biometeorol. 2023, 67, 957. 37060454 10.1007/s00484-023-02467-7 PMC10267279

[11] Y. Ojima , S. Torii , Y. Maeda , A. Matsuura , Animals. 2022, 12, 2505. 36230247 10.3390/ani12192505 PMC9559210

[12] M. A. Brownlow , J. X. Mizzi , Equine. Vet. Educ. 2022, 34, 259.

[13] R. P. Rhoads , L. H. Baumgard , J. K. Suagee , S. R. Sanders , Adv. Nutr. 2013, 4, 267. 23674792 10.3945/an.112.003376 PMC3650495

[14] M. Saeed , G. Abbas , M. Alagawany , A. A. Kamboh , M. E. Abd El‐Hack , A. F. Khafaga , S. Chao , J. Therm. Biol. 2019, 84, 414. 31466781 10.1016/j.jtherbio.2019.07.025

[15] A. R. Thompson , T. Jones , K. A. Guay , J. L. Leatherwood , J. Anim. Sci. 2019, 97, 31.

[16] Y. Ling , T. An , L. W. Yap , B. Zhu , S. Gong , W. Cheng , Adv. Mater. 2020, 32, 1904664. 10.1002/adma.201904664 31721340

[17] J. R. Sempionatto , V. R.‐V. Montiel , E. Vargas , H. Teymourian , J. Wang , ACS Sens. 2021, 6, 1745. 34008960 10.1021/acssensors.1c00553

[18] Y. Wang , C. Zhao , J. Wang , X. Luo , L. Xie , S. Zhan , J. Kim , X. Wang , X. Liu , Y. Ying , Sci. Adv. 2021, 7, eabe4553. 33523953 10.1126/sciadv.abe4553 PMC10964967

[19] J. Zhou , S. Zhou , P. Fan , X. Li , Y. Ying , J. Ping , Y. Pan , Nano‐Micro Lett. 2024, 16, 49. 10.1007/s40820-023-01274-4 PMC10716106 38087121

[20] M. Bariya , H. Y. Y. Nyein , A. Javey , Nat. Electron. 2018, 1, 160.

[21] J. Min , J. Tu , C. Xu , H. Lukas , S. Shin , Y. Yang , S. A. Solomon , D. Mukasa , W. Gao , Chem. Rev. 2023, 123, 5049. 36971504 10.1021/acs.chemrev.2c00823 PMC10406569

[22] M. Bariya , H. Y. Y. Nyein , A. Javey , Nat. Electron. 2018, 1, 160.

[23] D. R. Seshadri , R. T. Li , J. E. Voos , J. R. Rowbottom , C. M. Alfes , C. A. Zorman , C. K. Drummond , NPJ. Digit. Med. 2019, 2, 72. 31341957 10.1038/s41746-019-0150-9 PMC6646404

[24] P. C. Ferreira , V. N. Ataíde , C. L. Silva Chagas , L. Angnes , W. K. Tomazelli Coltro , T. R. Longo Cesar Paixão , W. Reis de Araujo , Trends. Anal. Chem. 2019, 119, 115622.

[25] T. Saha , R. Del Caño , K. Mahato , E. De la Paz , C. Chen , S. Ding , L. Yin , J. Wang , Chem. Rev. 2023, 123, 7854. 37253224 10.1021/acs.chemrev.3c00078

[26] H. Teymourian , M. Parrilla , J. R. Sempionatto , N. F. Montiel , A. Barfidokht , R. Van Echelpoel , K. De Wael , J. Wang , ACS Sens. 2020, 5, 2679. 32822166 10.1021/acssensors.0c01318

[27] Y. Song , J. Min , Y. Yu , H. Wang , Y. Yang , H. Zhang , W. Gao , Sci. Adv. 2020, 6, eaay9842. 32998888 10.1126/sciadv.aay9842 PMC7527225

[28] Y. Yamamoto , S. Harada , D. Yamamoto , W. Honda , T. Arie , S. Akita , K. Takei , Sci. Adv. 2016, 2, e1601473. 28138532 10.1126/sciadv.1601473 PMC5262446

[29] M. Parrilla , I. Ortiz‐Gómez , R. Cánovas , A. Salinas‐Castillo , M. Cuartero , G. A. Crespo , Anal. Chem. 2019, 91, 8644. 31194514 10.1021/acs.analchem.9b02126

[30] M. Parrilla , M. Cuartero , G. A. Crespo , TRAC‐Trend. Anal. Chem. 2019, 110, 303.

[31] F. Gao , C. Liu , L. Zhang , T. Liu , Z. Wang , Z. Song , H. Cai , Z. Fang , J. Chen , J. Wang , M. Han , J. Wang , K. Lin , R. Wang , M. Li , Q. Mei , X. Ma , S. Liang , G. Gou , N. Xue , Microsyst. Nanoeng. 2023, 9, 1. 36597511 10.1038/s41378-022-00443-6 PMC9805458

[32] Y. Yang , Y. Song , X. Bo , J. Min , O. S. Pak , L. Zhu , M. Wang , J. Tu , A. Kogan , H. Zhang , T. K. Hsiai , Z. Li , W. Gao , Nat. Biotechnol. 2020, 38, 217. 31768044 10.1038/s41587-019-0321-x

[33] H. Liu , Z. Sun , Y. Chen , W. Zhang , X. Chen , C.‐P. Wong , ACS Nano. 2022, 16, 10088. 35786945 10.1021/acsnano.2c02812

[34] F. M. Vivaldi , A. Dallinger , A. Bonini , N. Poma , L. Sembranti , D. Biagini , P. Salvo , F. Greco , F. Di Francesco , ACS Appl. Mater. Interfaces. 2021, 13, 30245. 34167302 10.1021/acsami.1c05614 PMC8289247

[35] J. Zhu , X. Huang , W. Song , ACS Nano. 2021, 15, 18708. 34881870 10.1021/acsnano.1c05806

[36] F. Zhao , J. He , X. Li , Y. Bai , Y. Ying , J. Ping , Biosens. Bioelectron. 2020, 170, 112636. 33017772 10.1016/j.bios.2020.112636

[37] J. Tu , J. Min , Y. Song , C. Xu , J. Li , J. Moore , J. Hanson , E. Hu , T. Parimon , T.‐Y. Wang , E. Davoodi , T.‐F. Chou , P. Chen , J. J. Hsu , H. B. Rossiter , W. Gao , Nat. Biomed. Eng. 2023, 7, 1293. 37349389 10.1038/s41551-023-01059-5 PMC10592261

[38] M. Wang , Y. Yang , J. Min , Y. Song , J. Tu , D. Mukasa , C. Ye , C. Xu , N. Heflin , J. S. McCune , T. K. Hsiai , Z. Li , W. Gao , Nat. Biomed. Eng. 2022, 6, 1225. 35970928 10.1038/s41551-022-00916-z PMC10432133

[39] A. R. Cardoso , A. C. Marques , L. Santos , A. F. Carvalho , F. M. Costa , R. Martins , M. G. F. Sales , E. Fortunato , Biosens. Bioelectron. 2019, 124, 167. 30388558 10.1016/j.bios.2018.10.015

[40] G. Zhao , F. Wang , Y. Zhang , Y. Sui , P. Liu , Z. Zhang , C. Xu , C. Yang , Appl. Surf. Sci. 2021, 565, 150565.

[41] A. C. Marques , A. R. Cardoso , R. Martins , M. G. F. Sales , E. Fortunato , ACS Appl. Nano. Mater. 2020, 3, 2795.

[42] Z. Chu , C. Liu , Y. Lu , Mater. Lett. 2022, 323, 132537.

[43] C. Hou , Q. Luo , Y. He , H. Zhang , J. Appl. Electrochem. 2021, 51, 1721.

[44] Y. Zhao , Y. Yu , S. Zhao , R. Zhu , J. Zhao , G. Cui , Microchem. J. 2023, 185, 108092.

[45] Y. Gai , E. Wang , M. Liu , L. Xie , Y. Bai , Y. Yang , J. Xue , X. Qu , Y. Xi , L. Li , D. Luo , Z. Li , Small Methods. 2022, 6, 2200653. 10.1002/smtd.202200653 36074976

[46] Q. Shao , G. Liu , D. Teweldebrhan , A. A. Balandin , Appl. Phys. Lett. 2008, 92, 202108.

[47] M. Yang , N. Sun , X. Lai , J. Wu , L. Wu , X. Zhao , L. Feng , ACS Sens. 2023, 8, 176. 36604942 10.1021/acssensors.2c02016

[48] S. Erbas‐Cakmak , S. Kolemen , A. C. Sedgwick , T. Gunnlaugsson , T. D. James , J. Yoon , E. U. Akkaya , Chem. Soc. Rev. 2018, 47, 2228. 29493684 10.1039/c7cs00491e

[49] I. Belhadj Slimen , T. Najar , A. Ghram , M. Abdrrabba , J. Anim. Physiol. Anim. Nutr. 2016, 100, 401. 10.1111/jpn.12379 26250521

[50] H. Kang , R. R. Zsoldos , A. Sole‐Guitart , E. Narayan , A. J. Cawdell‐Smith , J. B. Gaughan , Int. J. Biometeorol. 2023, 67, 957. 37060454 10.1007/s00484-023-02467-7 PMC10267279

[51] L. Mo , X. Ma , L. Fan , J. H. Xin , H. Yu , Chem. Eng. J. 2023, 454, 140473.

[52] W. Gao , S. Emaminejad , H. Y. Y. Nyein , S. Challa , K. Chen , A. Peck , H. M. Fahad , H. Ota , H. Shiraki , D. Kiriya , D.‐H. Lien , G. A. Brooks , R. W. Davis , A. Javey , Nature. 2016, 529, 509. 26819044 10.1038/nature16521 PMC4996079

