# Supplementary material for: A laser‐Engraved Wearable Electrochemical Sensing Patch for Heat Stress Precise Individual Management of Horse
Source: Adv Sci (Weinh). 2024 May 10;11(28):2310069. doi: 10.1002/advs.202310069 (PMC11267262; doi:10.1002/advs.202310069)
Supplement: Supplementary file 1 — Supporting Information [file ADVS-11-2310069-s001.pdf]

ADVANCED SCIENCE
Open Access

Supporting Information

for *Adv. Sci.*, DOI 10.1002/advs.202310069

A laser-Engraved Wearable Electrochemical Sensing Patch for Heat Stress Precise Individual Management of Horse

*Yuxiang Pan, Xiaoyu Su, Ying Liu, Peidi Fan, Xunjia Li, Yibin Ying and Jianfeng Ping**

Supporting Information for

A laser-engraved wearable electrochemical sensing patch for heat stress precise individual management of horse

*Yuxiang Pan, Xiaoyu Su, Ying Liu, Peidi Fan, Xunjia Li, Yibin Ying, and Jianfeng Ping**

Y. Pan, X. Su, Y. Liu, P. Fan, Y. Ying, J. Ping

Laboratory of Agricultural Information Intelligent Sensing

College of Biosystems Engineering and Food Science

Zhejiang University, Hangzhou, 310058, PR China

E-mail: jfping@zju.edu.cn

Y. Pan, X. Su, X. Li, Y. Ying, J. Ping

ZJU-Hangzhou Global Scientific and Technological Innovation Center

Zhejiang University, Hangzhou, 311215, PR China

Index

S1. Reagents ……………………..…………………………………………………..3

Figure S1 ..……………………………………...……………..…………………4

Figure S2 ……………………………………………………….…………………4

Figure S3 ……………………………………………………….…………………5

Figure S4 ……………………………………………………….………….………5

Figure S5 ……………………………………………………….………….………6

Figure S6 ………………………………………………………………….………6

Figure S7 ……………………………………………………….………….………7

Figure S8 ……………………………………………………….…………………7

Figure S9 ……………………………………………………….…………………8

Figure S10 ………………………………………………….………….…………..8

Figure S11 ………………………………………………….…………….………..9

Figure S12 ………………………………………………….…………..…………..9

Figure S13 ………………………………………………….………..……………..10

Table S1 ………………………………………………………………………….…….11

Table S2 ………………………………………………………………………….…….12

References

(Supplementary) ……………………………………………………………...13**S1. Reagents**

Sodium chloride (NaCl), potassium monochloride (KCl), aniline, silver nitrate ($AgNO_3$), sodium thiosulfate ($Na_2S_2O_3$), sodium bisulfite ($NaHSO_3$), polyvinyl butyral (PVB), methanol, agarose, carbachol, 3,4-ethylenedioxythiophene (EDOT), poly(sodium 4-styrenesulfonate) (NaPSS), iron (III) chloride ($FeCl_3$), potassium ferricyanide (III), potassium ferricyanide ($K_3[Fe(CN)_6]$), and potassium hexacyanoferrate trihydrate $K_4Fe(CN)_6{\cdot}3H_2O$ were provided by Shanghai Macklin Biochemical Co. Ltd. (Shanghai, China). Bis(2-ethylehexyl) sebacate (DOS), high-molecular-weight polyvinyl chloride (PVC), valinomycin, sodium tetraphenylborate (NaTPB), polyvinyl butyral (PVB), carbachol, polyaniline (PANI), and cyclohexanone were purchased from Beijing Chemical Reagent Co. Ltd. (Beijing, China). A polyimide film (75 μm) was were purchased from DuPont China Holding Co., Ltd. (Beijing, China). In entire experiments, deionized water (>18.2 MΩ.cm) were supplied by Milli-Qultrapure. Other reagents were purchased from Aladdin Industrial Co. Ltd. (Shanghai, China).

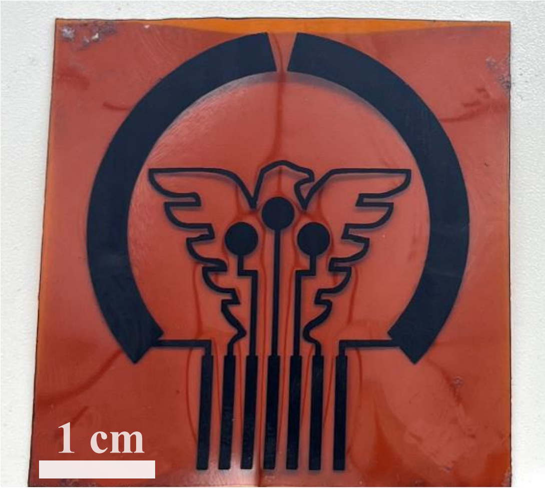

Figure S1. Dimensional diagram of the LEG-based wearable patch.

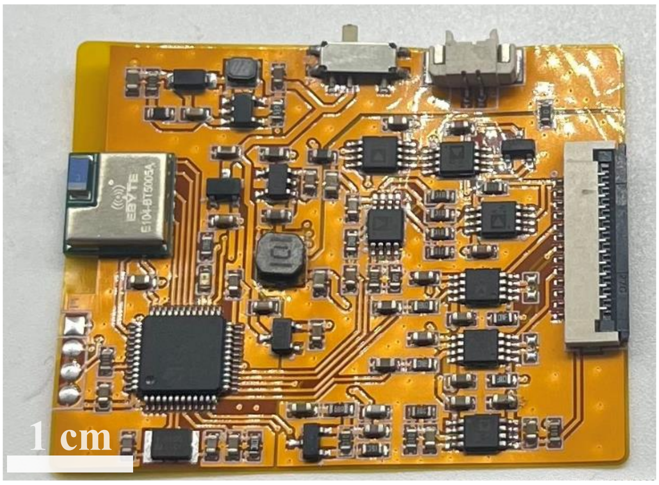

Figure S2. Dimensional diagram of FPCB.

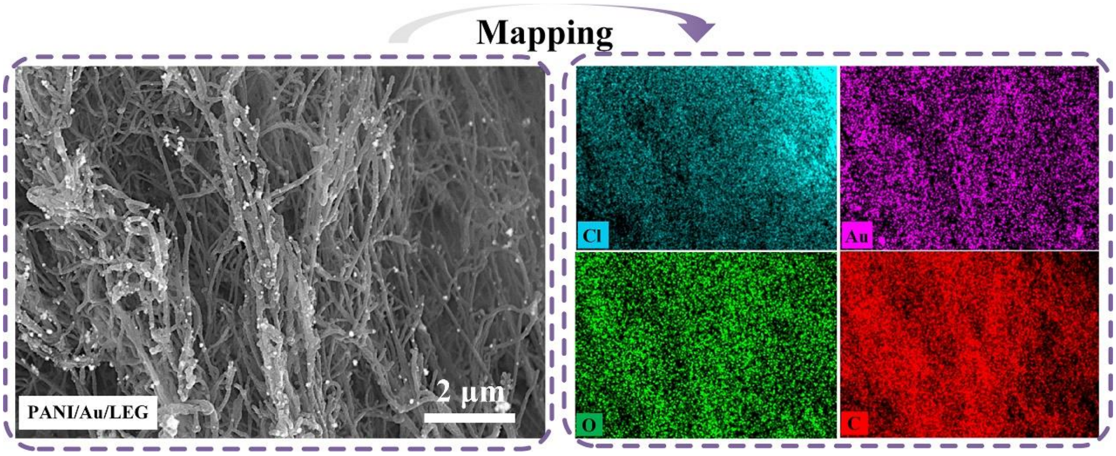

Figure S3. SEM and mapping images of PANI/Au/LEG. Elements of Cl, Au, C, and O are evenly dispersed on the surface of PANI/Au/LEG.

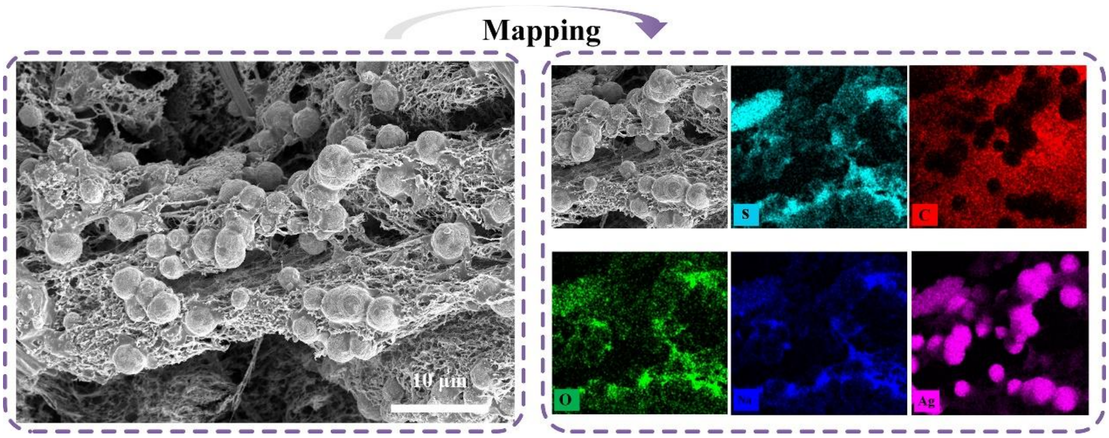

Figure S4. SEM and mapping images of Ag/LEG. Elements of S, C, O, Na, and Ag are evenly dispersed on the surface of Ag/LEG.

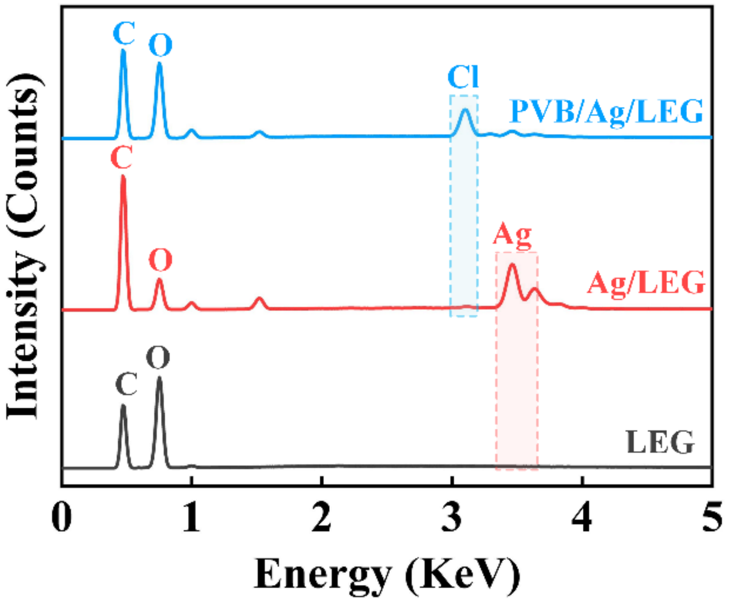

Figure S5. EDS spectra of bare LEG, Ag/LEG, and PVB/Ag/LEG. Ag element is appeared on the Ag/LEG and the Cl element is appeared on the PVB/Ag/LEG.

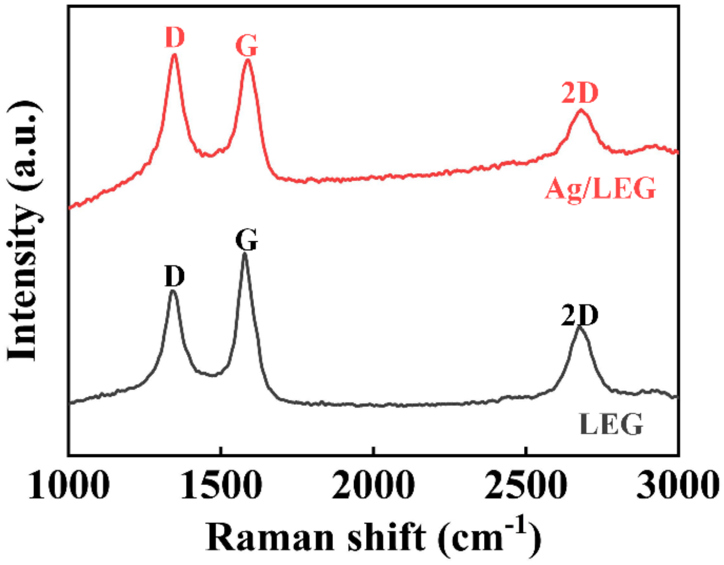

Figure S6. Raman spectra of bare LEG and Ag/LEG. The value of I_D/I_G is obviously changed after Ag modification.

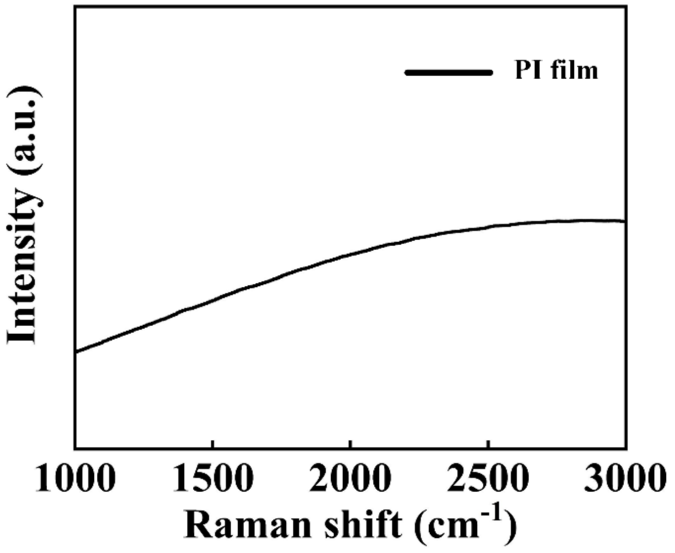

Figure S7. Raman spectra of pure PI substrate. There are no characteristic peaks on pure PI substrate.

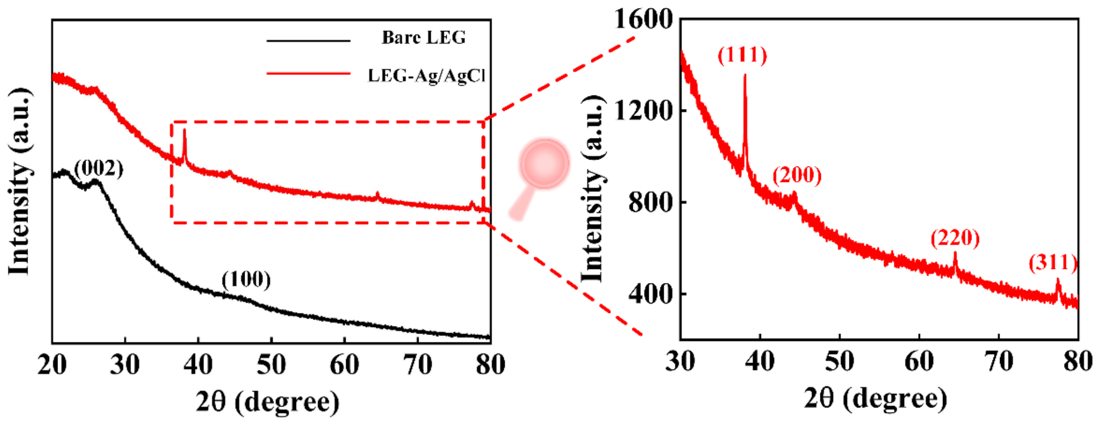

Figure S8. XRD patterns of bare LEG and LEG-Ag/AgCl. Bare LEG presented characteristic crystal faces of (002) and (100). For Ag/LEG, the representative crystal faces of Ag are appeared of (111), (200), (220), and (311).

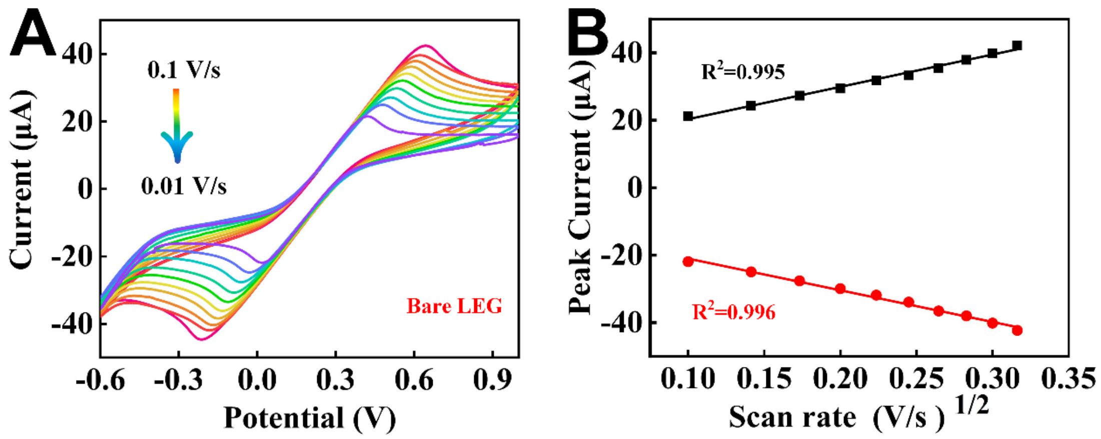

Figure S9. (A) CVs and (B) calibration plots (redox peaks vs. square roots of scan rates) of bare LEG in 5 mM $K_3[Fe(CN)_6]$ and 0.1 M KCl. Following increasing scan rates as 0.01~0.1 V/s (step: 0.01 V/s), bare LEG presents similar upward tendencies.

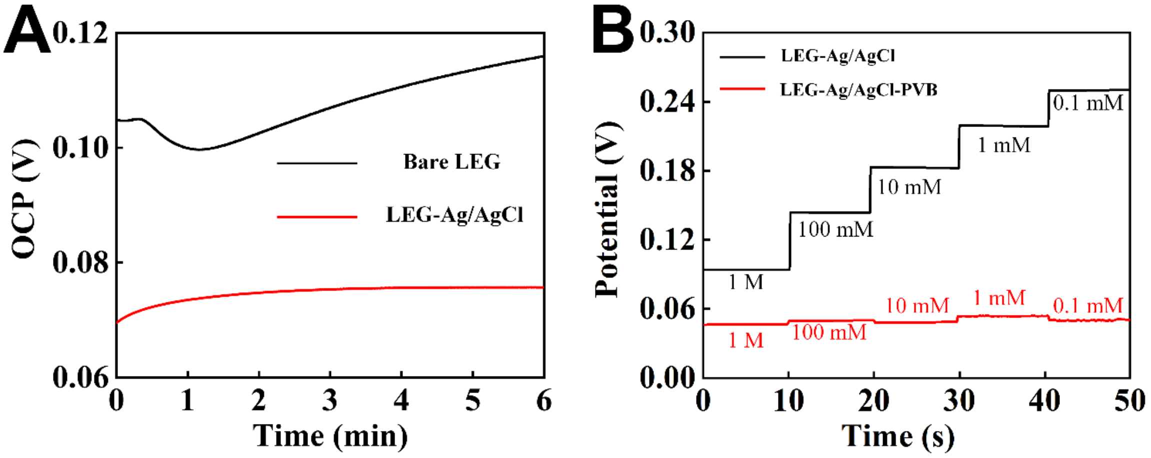

Figure S10. (A) OCP responses of bare LEG and LEG-Ag/AgCl. (B) Stability exploration of LEG-Ag/AgCl and LEG-Ag/AgCl-PVB in different NaCl.

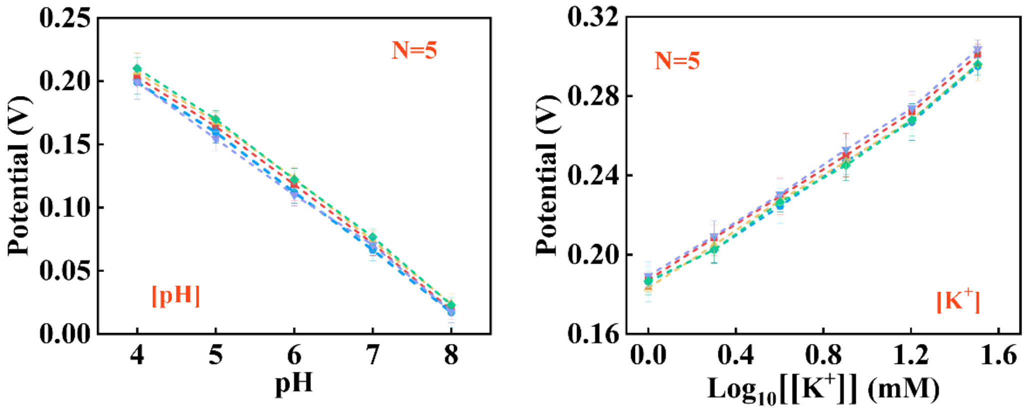

Figure S11. Reproducibility of (A) pH and (B) K^+ sensors. Five sweat sensors presented similar potential responses.

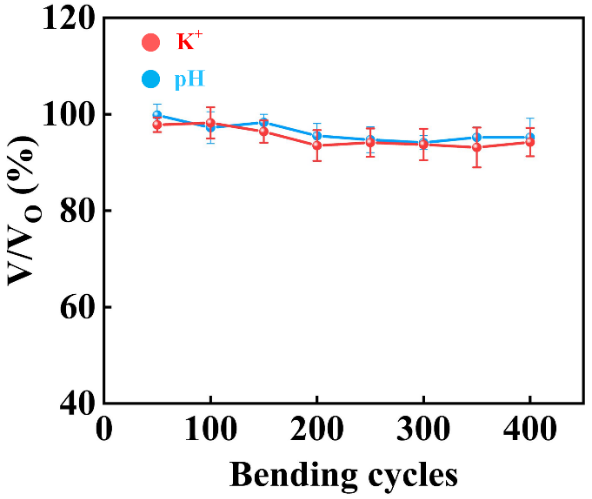

Figure S12. Potential variation of the pH and K^+ sensors before and after bending, where the V and V_0 are represent the potential before and after bending, respectively. The sensor presented satisfactory durability after 400 bending cycles.

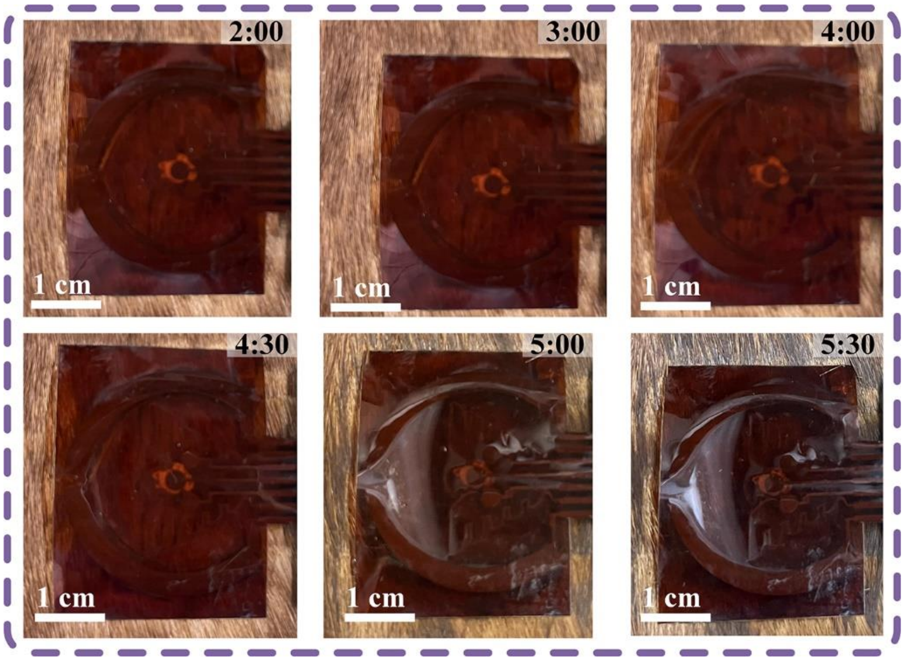

Figure S13. Flexible sensor patches with sweat induce module is covered on the surface

of the horse. As a high-value animal, the hair of horse is thick and cannot be completely shaved, which likely leads to water loss in the horse[1, 2]. Different from humans, the efficiency of sweat-stimulation in-body sweat analysis of horse is not ideal, so the matching degree between sweat-stimulation system and animal skin needs promoted**Table S1.** The comparison of recently reported sweat sensors for pH, K^+, and temperature determination, where the *S* represents sensitivity of sensor.

Analytical method	*S* for pH (mV/dec)	*S* for K^+ (mV/dec)	Temperature sensing	Suitable for wearable sensors	Ref.
Electrochemical	-	58.0	×	√	[3]
Electrochemical	54.0	56.0	×	√	[4]
Electrochemical	71.86	-	×	√	[5]
Electrochemical	69.33	-	×	×	[6]
Electrochemical	-	34.7	×	√	[7]
Electrochemical	-	60.06	×	×	[8]
Electrochemical	-	61.3	√	√	[9]
Electrochemical	63.8	60.3	√	√	This work

Table. S2. Method comparison for sweat (a) pH and (b) K^+ concentrations measurement with our sensors and standard method.

(a) Species (N=3)		Method comparison		
		This method	pH meter	|Error|
pH	Sample 1	6.29±0.012	6.04±0.009	4.1%
	Sample 2	4.45±0.006	4.31±0.008	3.2%
	Sample 3	5.38±0.008	5.76±0.012	6.6%
(b) Species (N=3)		Method comparison (mM)		

		This method	ICP-MS	|Error|
	Sample 1	4.36±0.006	4.12±0.004	5.8%
K^+	Sample 2	7.63±0.015	7.94±0.007	3.9%
	Sample 3	3.21±0.005	3.35±0.003	4.2%

References

[1] C. Lovatt Evans, *British Veterinary Journal.* **1966**, 122, 3, 117.

[2] M. P. Szczepanik, P. M. Wilkołek, R. Adamek Ł, M. Gołyński, W. Sitkowski, I. Taszkun. *Polish Journal of Veterinary Sciences.* **2018**, 21, 35-38.

[3] J. Hao, Z. Zhu, C. Hu, Z. Liu, *Analytical Chemistry.* **2022**, 94, 4547.

[4] X. Cui, Y. Bao, T. Han, Z. Liu, Y. Ma, Z. Sun, *Talanta.* **2022**, 245, 123481.

[5] Z. Xu, X. Qiao, R. Tao, Y. Li, S. Zhao, Y. Cai, X. Luo, *Biosensors and Bioelectronics.* **2023**, 234, 115360.

[6] Y. Zhao, Y. Yu, S. Zhao, R. Zhu, J. Zhao, G. Cui, *Microchemical Journal.* **2023**, 185, 108092.

[7] L. Mo, X. Ma, L. Fan, J. H. Xin, H. Yu, *Chemical Engineering Journal.* **2023**, 454, 140473.

[8] J. Wei, X. Zhang, Q. Chang, S. M. Mugo, Q. Zhang, *Analytical Chemistry.* **2023**, 42, 95, 15786.

[9] W. Gao, S. Emaminejad, H. Y. Y. Nyein, S. Challa, K. Chen, A. Peck, H. M. Fahad, H. Ota, H. Shiraki, D. Kiriya, D.-H. Lien, G. A. Brooks, R. W. Davis, A. Javey, *Nature.* **2016**, 7587, 509.
